# Learning to read FAST MRI: Qualitative interviews with groups experienced reading mammograms

**DOI:** 10.3310/nihropenres.13817.2

**Published:** 2025-04-24

**Authors:** Sam Harding, Rebecca Geach, Lyn Jones, Fast MRI Study Group

**Affiliations:** 1 North Bristol NHS Trust, Westbury on Trym, England, UK; 2 University of Bristol School of Oral and Dental Sciences, Bristol, England, UK

**Keywords:** Breast screening, FAST MRI, Abbreviated breast MRI, Qualitative, COM-B, Implementation.

## Abstract

**Objectives:**

Abbreviated breast MRI (abMRI) is being introduced into breast screening practice worldwide. Increased provision of abMRI will require mammogram readers to learn abMRI-interpretation and the implementation of abMRI-reading into clinical practice. The present study explores the acceptability of the implementation of developed reader training, and the barriers and facilitators to training programme participation and subsequently to reading the training programme assessment task of abMRI images in a work/NHS context familiar to the individual participants.

**Methods:**

Fourteen NHS Breast Screening Programme mammogram readers, who were undertaking abMRI interpretation training, participated in semi-structured interviews. Template analysis using the a priori implementation framework, COM-B (Capability, Opportunity, Motivation, Behaviour) was undertaken.

**Results:**

The training day was well received. Participants identified that their varying ranges of knowledge and experience (capability) was accounted for. Participation in the research was appreciated by all, but especially those new to reading MRI.

Radiographers commented that learning to read and understand the abMRI images was motivational, and this helped drive implementation. It was noted that organisational leadership is needed to fully enable change in practice. COVID-19 was commented on in relation to its impact on image reading.

**Conclusions:**

The project demonstrates that production of training for reading abMRI images and subsequent implementation of changes to practice needs to be carefully planned. Changes must be led by the needs of staff undertaking the tasks. When this is achieved the engagement in training is positive and the barriers are more readily removed or mitigated for both individuals and organisations.

**Critical relevance statement:**

AbMRI is part of the solution to reducing waiting times for MRI within the NHS, however, training for reading abMRI images and implementation to practice needs to be carefully planned.

## Plain language summary

**Objectives:**

A short version of an MRI called an abbreviated breast MRI (abMRI) is being introduced into breast screening practice worldwide. To achieve this in the NHS, staff that current interpret mammograms will have to learn to do the same for abMRI. The present study explores how acceptable a training programme developed to teach these skills is to staff, and to look at the barriers and facilitators to training programme participation and subsequently to interpreting the training programme assessment task of abMRI images in a work/NHS context familiar to the individual participants.

**Methods:**

Fourteen NHS Breast Screening Programme mammogram readers, who were undertaking abMRI interpretation training, participated in semi-structured interviews. The interview data was assessed by using a methodology called ‘Template Analysis’. This uses a pre-existing framework, to map factors onto, in this study it was the COM-B (Capability, Opportunity, Motivation, Behaviour).

**Results:**

The training day was well received. Participants said that their varying ranges of knowledge and experience (capability) was accounted for by the content and the delivery style of the day. Participation in the research was appreciated by all, but especially those new to reading MRI. Radiographers commented that learning to read and understand the abMRI images was motivational, and this helped drive implementation. It was noted that organisational leadership is needed to fully enable change in practice. COVID-19 was commented on in relation to its impact on image reading and interpretation.

**Conclusions:**

The project demonstrates that production of a training programme for reading abMRI images and subsequent implementation of changes to practice needs to be carefully planned. Changes must be led by the needs of staff undertaking the tasks. When this is achieved the engagement in training is positive and the barriers are more readily removed or mitigated for both individuals and organisations.

## Key points

1. Upscaling abbreviation MRI (abMRI) provision requires the workforce of mammogram readers to be able both learn and implement the reading of the abMRI images.
2. The principles of implementation science must be combined with pragmatic understanding of individual and organisational level barriers to produce acceptable and usable training and process change.
3. A single day training can be accessed by clinical staff and made acceptable through consultation regarding venue and timing.

## Abbreviations

abMRI            abbreviated breast Magnetic Resonance Imaging

COM-B          Capability, Opportunity, Motivation, Behaviour

FAST MRI     first post-contrast subtracted images Magnetic Resonance Imaging

fpMRI            full-protocol breast MRI

IS                    Implementation science

MIP                 maximum-intensity projection

MRI                Magnetic Resonance Imaging

NHSBSP         National Health Service Breast Screening Programme

PACS              Picture Archiving and Communication System

SpA                 supporting professional activities

## Introduction

Breast cancer screening saves lives by finding breast cancers early ^1^. Screening with mammography is cost effective but imperfect, as it results in over-diagnosis and under-diagnosis ^2, 3^. It also uses ionising radiation and carries a small risk of radiation-induced breast cancer ^4^. An ideal screening test for breast cancer would be able to selectively detect aggressive cancers without detecting those that if left without treatment would not cause harm during a woman’s lifetime. It would also not use ionising radiation, be acceptable to screening clients, and no more burdensome than mammography to acquire or to report. Abbreviated MRI (abMRI) is proposed as a screening tool for breast cancer that could prove more cost effective than mammograms for some women ^5– 7^. However, its feasibility, acceptability, cost effectiveness and its effect on overdiagnosis remains to be defined through research. 

Learning to read and interpret full-protocol breast MRI (fpMRI) is a significant process, taking a number of years to achieve accreditation ^8– 10^. However, a previous single-centre study, of 8 readers from the UK National Health Service Breast Screening Programme (NHSBSP), suggested that NHSBSP mammogram readers could be effectively trained to interpret abbreviated MRI (abMRI) protocol with a single day’s training ^11, 12^. This has been supported by a larger study covering 6 sites and 37 readers ^13^. A simplified form of abMRI (first post-contrast subtracted images (FAST MRI), is displayed as maximum-intensity projection (MIP) and subtracted slice stack, and this is the form used in the current study. 

Integration of new imaging test into the existing NHSBSP framework requires the multi-professional workforce of readers of all levels of experience with breast MRI to undergo additional training ^14^. However, the ability of the workforce to effectively read abMRI does not mean that services can be implemented in practice.

Implementation science (IS) is a crucial framework in the development of tools within healthcare environments. As healthcare institutions strive to integrate technological advancements, the principles of IS offer a systematic approach to address the unique challenges in this complex setting. The exploration of factors such as organisational culture, clinician readiness, and workflow integration becomes paramount in ensuring the successful implementation. This perspective aligns with the works of Proctor *et al*. ^15^ and Nilsen ^16^, who emphasise the significance of understanding contextual factors and the need for tailored implementation strategies. By employing IS methodologies, developers can navigate the intricate landscape of healthcare systems, align tools with clinical workflows, and address the diverse needs of professionals. This facilitates the effective adoption of educational technologies and contributes to improved patient outcomes. The incorporation of IS principles in the development of learning tools for healthcare environments is pivotal for realising the transformative potential of technology ^15, 16^. The authors of the present study explored the acceptability of the implementation of the developed reader training, the barriers and facilitators to participating in the training programme and reading the abMRI images in a NHS context. The success of the training is reported elsewhere ^11, 13^.

## Methods

### Patient and public involvement

Patient and public involvement is at the heart of the FAST MRI programme of work, although patients were not involved in the development or design of this work. Wider stakeholder engagement in the form of Radiologist and Radiographers, not part of the current project, were collaborated with in relation to deciding on the data collection methodology and the content of the interview schedule.

This work is a sub-study of an ethically approved programme of work (REC: 19/LO/1473 and IRAS:258203). Participants from the main study were additionally consented to participant in the interviews undertaken and reported herein.

### Training context

NHSBSP multi-professional mammogram readers, fully qualified to interpret mammograms ^17^, at 6 sites (NHSBSP screening units) within the South-West Region of England were invited to participate (5 Advanced practitioners, 3 Breast Clinicians, 5 Consultant Radiologists, 1 Radiographer). One group interpreted fpMRI in their routine clinical practice, and another group did not. Participants attended 1 of eight single days of face-to-face standardised training, and then interpreted a test set of abMRI scans. Interviews were undertaken within 1 week of the participants completion of reading the test sets, but prior to any feedback on accuracy of their judgements.

### Participants

Fourteen interviews were undertaken (Group 1, n=5 Consultant Radiologists, and Group 2 n=9; Advanced Practitioner Radiographers and Breast Clinicians). These interviews were conducted via Zoom. All interviews were undertaken by the primary author, and no prior relationship existed between her and any of the interviewees. All interviewees were in their workplace at the time of the interview, as was the interviewer. No payment or other gratuity was made to the participants of these interviews.

A semi-structured interview schedule was developed with stakeholders from previous research ^12^.

### Analysis

Template analysis was applied to the transcripts of the interviews. The Template method supports a hybrid approach of both deductive (a priori) and inductive qualitative analysis. In this case using an a priori implementation framework whilst remaining open to the possibility that the framework is not complete or completely implementable within an acute NHS trust ^18, 19^. 

Following transcription of the interviews transcripts were uploaded to NVivo 12 ^20^, and the King & Brooks ^19^ template process undertaken:

***Stage 1: Transcription***

***Stage 2: Familiarisation with the interview***

***Stage 3: Coding***

***Stage 4: Development or selection of a working analytical framework***

The a priori framework selected for use from this stage of analysis was the COM-B ^21^. COM-B is an abbreviation of ‘Capability, Opportunity, Motivation, Behaviour’ and is a model relating to the theory of behaviour ^21^. COM-B posits behaviour as the result of an interaction between three components: capability, opportunity, and motivation and is used in IS research as well as behaviour change. 

***Stage 5: Applying the analytical framework with consideration of additional inductive codes***

Coding patterns were considered in light of the COM-B framework with tentative themes being redefined or discarded.

***Stage 6: Charting data into the framework matrix***

Refined codes were then mapped against the a priori framework, and additional themes were retained for inductive inclusion in the interpretation of the data.

***Stage 7: Interpreting the data ^19, 22^***

This analysis was primarily completed by the fist author, who is a health psychologist and senior research fellow with significant experience with qualitative research and IS. The coding was reviewed by the second and third authors, both of whom are consultant radiologists.

## Results

A self-selecting subset of participants in an abMRI interpretation training study previously reported in 13 were interviewed. The 14 participants had been NHSBSP mammogram readers for an average 8.8 years (Range 1 year to 22 years). All but one participant were female. Interviews lasted between 15:52 and 46:02 minutes (average 34:25 minutes)

### Training day

A challenge recognised by the research team was that of the range of (breast MRI) experience of attendees (capability). It was also critical that the participants were able to attend the training day and that the physical arrangements (opportunity) were such that attendance was easy for them.

***Capability***

All participants commented on how knowledge and understanding were developed and built on from basic to advanced levels, and this was appreciated by those that were both new to reading MRI and those with more experience.

*‘For me obviously with years of experience in MRI there were some bits that I didn’t need but I think that just thinking about the very diverse needs of everybody attending it, I thought it was extremely well organised actually and very worthwhile.’* (RG_46)

***Opportunity***

In the development of the training days, we consulted potential participants about what factors were important to maximise the chances of attending the training. This was reflected on during the interviews.

The physical location of the training was important to the recruits. Providing a full day’s training outside the participants’ place of work was required, being able to start later than a normal working day, to enable a longer journey than normal (commute) and finish early was appreciated.

*‘The parking, everything, all the organisation side of things went really, really smoothly. The lectures were really interesting, so I knew exactly what my role was in the study and what I was meant to be doing and they went through, like you just have, all the consent and everything was really clear, and then the room and the set up and being able to work on a workstation, the coffee breaks were a little bit long, but lunch was great, and everything being hand on more, I got a lot from it.’* (AG_26)

Ensuring sufficient staffing to enable breast clinics to continue at the participant’s NHS trust was of consideration from the individuals’ perspectives and for those who organised services. They reported that being able to collaborate with the research team to match their different trusts work patterns with training dates made it much easier to take part.

*‘One of the great things was being able to tell the research team what the services challenges are. Being asked for this type of information right from when the project was being planned [prior to obtaining funding] made it possible to plan my own attendance as well as when other staff could be available.’* (RP_08)

***Motivation***

Although not specifically a motivation for attending the training, there was an appreciation of the considerations made in relation to location, timing, parking, refreshment breaks and supplying a good lunch.

### Implementation

Following the training, there was an unexpected gap before the participants were able to access the training images (median 3.5 months (range 1.8 – 8.9). This is reflected in the participants’ discussions about their capability to undertake the activity.

***Capability***

The gap between training and implementation meant that participants felt they needed to revisit the training, and the materials supplied to them during the training day

*‘training day was a long time before we actually started doing them [reading assessment images] so by the time we actually got the images it was quite a distance so it was remembering what we were meant to be looking for? I know there were information sheets and I obviously went through all those but it’s not quite the same as when you’ve just done it on a course and it’s fresh in your memory.’* (AG_34)

It was recognised that some participants (radiologists and breast clinicians) would have dedicated SpA (supporting professional activities) time each week, and it was anticipated that these people would undertake reading of test images in this time. Whilst other participants, via their trusts, were offered to be compensated for the time it took them.

*‘It is always difficult finding the time to do a new task but knowing I could be reimbursed for the time was nice, especially when you know that some of your colleagues get time each week to undertake this type CPD activity and I don’t.’* (AC_17)

***Opportunity***

Physically, across the sites, the software was available on a limited number of Picture Archiving and Communication System (PACS) workstations, and these were required for specific clinical tasks, leading to reduced availability, as clinical reporting takes precedent to research. Due to software limitations and randomisation of image presentation, each participant was required to complete reading of all of the assessment images on the same workstation. So, participants had to wait or negotiate use of these machines or undertake the task outside of normal working hours when there was availability.

*‘The computers were in individual Radiologists offices and I am sure they would have been more than happy for us to go in and use that reading station, however, I think a lot of us felt we didn’t really want to park ourselves on a regular basis for long periods of time so I did tend to come in early, my children are a bit bigger so I can drop them at school quite early, so I came in early and tried to read 15 cases before I started work officially and that’s how I got through them.’* (AG_34)

Reading the test images coincided with the first wave of the COVID-19 pandemic. The participants had to deal with rapidly changing working patterns and workforce requirements. In the interviews the participants talked about how they could undertake reading the assessment images as well as the rapidly changing rules and regulations within the working environment.

*‘One day you were able to get into the office to read the images, and the next day only one person can be in the room, and obviously have to prioritise the clinical images over the research.’* (AG_27)

However, for those readers who had a compatible PACS workstation available at home, or whose organisation made this possible early during the pandemic, the change to work patterns and the reduction in clinically routine image reading associated with the national COVID-related pause in breast screening (March-May 2020) facilitated their engagement with the project.

*‘Yer know prior to Covid it's sort of you’re working flat out and working at 100 miles an hour, and now things aren't necessarily going at 100 miles an hour it's quite nice to sort of take the extra time to understand the teaching, review the notes and do the images’* (AN_23)

***Motivation***

The study team was in close contact with all the readers across the study, and there was appreciation that we were supportive and not overbearing. The participants noted that they *‘never felt under pressure at all to get it finished, everybody was very pleasant and just really grateful that we were doing it.’* (AG_21).

The radiographers all commented that learning to read and understand the abMRI images was highly motivational, especially as they were more able to understand MRI images shown during MDT meetings and participate in those discussions.

*‘Reading the images has transferred across to the day job because now when they show MRI pictures up in the MDT meeting my ears prick up and I look at it and they have started doing the scans and showing scans the way that the FAST MRI scan interprets it. Its really good to be able to understand and ask questions.’* (AC_17).

**Figure 1.**
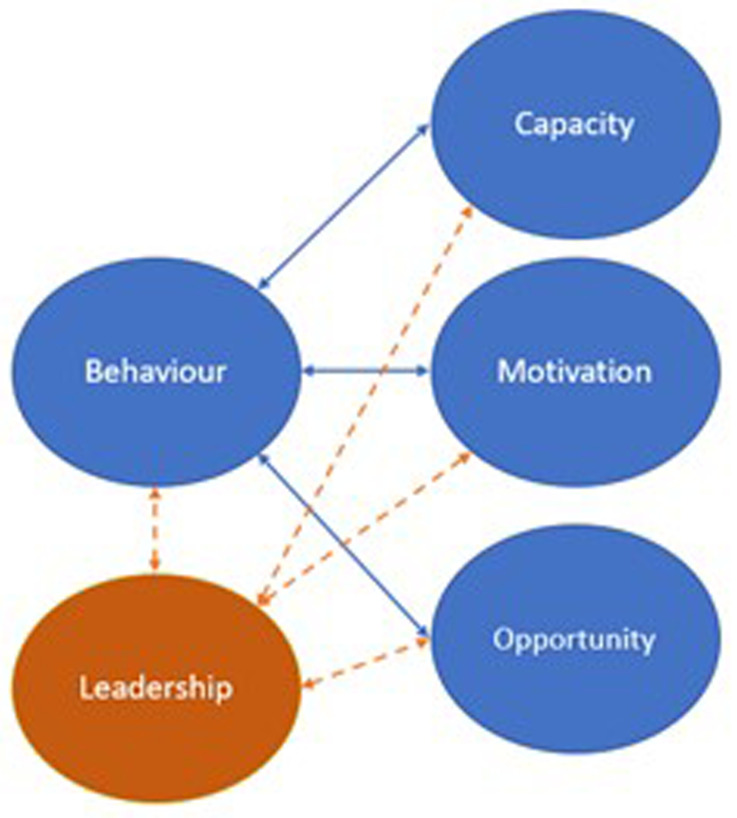
Coding tree of the template analysis.

Inductive coding led to one theme outside of the COM-B model being identified, that was relevant to both attending the training and being able to complete the training set. This theme was that of ‘leadership’. Figure 1 shows how leadership fits within the a priori framework of the COM-B used within the template analysis.

### Leadership

Going forward the readers indicated that the software and other resources, needed to be accessible in the same way that the current MRI reading setup is, with a recognition that to make this happen:

*‘service leads need to ‘buy-in’ to spending department money on new software and equipment, or at least to investing the time in training and subsequent updates and auditing of performance.’* (RC_14)
*‘You would need to be able to have it [the software] on radiologists personal workstations, and you’d need to be able to have their own personal workspaces, need to be within that workspace and probably integrated into the PACS system. But this would all depend on the department bosses Okaying it all.’* (RS_30)

It was suggested that being part of this research, future projects and potential service changes shows a desire of the service to demonstrate they are leaders in providing quality services of the community they serve.

*One of the organisational drivers to engage with this research and future stuff would be that if you said that it’s not just research and you made it clear that the plans is to implement a programme across the whole of the UK where MRI is done differently saving the NHS, you know, and also that it helps a current under-served community.* (RP_08)

## Discussion

The wider project, of which this work is part, was designed to assess if a single day abMRI interpretation training program for mammogram readers can achieve an overall diagnostic performance within benchmarks published for fpMRI ^13^. The focus of the current paper was to understand readers thoughts about the acceptability of the developed training programme (prior to being told they achievement on the training) and its implementation into practice. 

Using template analysis ^19^ with the a priori framework of the COM-B model ^21^ the recognition and preparation for the varying ranges of knowledge and experience of breast MRI (capability) of the participants was reported as being welcomed by all, making everyone feel included and able. The opportunity to be part of the research was appreciated by all participants, and especially by those new to reading MRI. But equally as important was that the training day was specifically tailored to fit into normal working hours, with accessible locations and free parking. The processes put in place to enable participation in the training are embedded in evidence-based recommendations to maximise training effectiveness ^23, 24^. The feedback from the currents study’s participants supports previous studies that have reported that well designed and implemented training provides benefits to individuals, teams, organisations, and society, including positive impact on organisational bottom-lines ^25^.

It is widely recognised that effectiveness of training needs to be assessed and this can be achieved in two keys ways. Firstly, are people able to successfully/correctly complete the tasks they have been trained to do, in real life settings, and secondly are the trainees are able to undertake the task (capable) and are they happy and able to do so (motivation and opportunity). The first of these issues is reported and addressed by Jones *et al.* ^13^. The second is addressed by the findings presented in the current paper.

As noted in the results section, the time between the training day and being able to implement the completion of the test set (summative assessment task) was longer than the researchers had planned. This led to participants feeling less able than they had immediately following their training, but they were self-motivated to revisit the materials supplied to them during the training day and reach out to the research team. 

It is relatively easy to look at the evidence-based methods to design training, but it is harder to implement across different healthcare services and organisations. It is recommended that after the training, physical obstacles are removed and appropriate tools and advice from the team are available, and that when problems did arise the trainees are happy to give rapid, open and honest feedback to the trainers (research team), so that they can amend and refine the implementation across all the sites ^26^. In the abMRI study this was undertaken by trying to optimise workstation access and support to find the time within a working day to undertake the required image reading. 

Overall, the nature of the leadership from within the research team, and the individual study sites (organisations) was recognised in the current project as vital. The trainees (participants) identified that going forward organisational leadership and championing of the change of practice will be needed to enable implementation of an abMRI national programme.

### Study limitations

This qualitative study was complimentary to a quantitative study where readers interpreted the test set of images on one occasion ^13^ and so we can provide no information on intra-reader variability.

We have not mapped people’s qualitative commentary on training and implementation with their previous professional image reading experience or their effectiveness at the image reading assessment. For this type of analysis to inform future implementation, a larger sample would be needed, and content analysis undertaken to code the qualitative data so that it can to some extent be quantitatively analysed with the effectiveness data. Additional detail may be available relating to implementation if this larger data set was subject to thematic analysis, however participation in the qualitative interview was a voluntary project within the wider quantitative work.

The wider quantitative work consisted of 37 participants. All were invited to participate in the interviews, however only 14 did. A range of reasons were provided for not being available including retiring from practice (n=3), general workload (n=12), maternity leave (n=2), sickness (n=4), and (n=2) administrative error where clinic was not cancelled, and therefore were unavailable for interview. It is possible that this biased the results, however, review of the interpretation of the interviews by the FAST MRI Study Group suggested an accurate understanding.

The study was complicated by spanning the first wave of the COVID-19 pandemic. Research participants had very different experiences of work including redeployment over this time period, it is possible that implementation of this work was actually easier for some, as fewer people were using the limited resources (PACS workstations) at core times, so readers could access the images when it suited them.

## Conclusions

Template analysis using the a priori framework of the COM-B, has proven to be a structured and effective way to understand the barrier and facilitators to the implementation of a developed abMRI training and assessment package. The project has demonstrated that production of training and the subsequent implementation of changes to practice can be undertaken in a way that is acceptable to individuals and services but must be led by the needs of the staff, in this specific case those reading the abMRI images. 

## Ethics approval and consent to participate

This work is a sub-study of an ethically approved programme of work assessed by London‐Bromley Research Ethics Committee (REC: 19/LO/1473) and by the Health Research Authority and Health and Care Research Wales (IRAS:258203). 

Participants from the main study provided additional written consent to participant in the interviews undertaken and reported herein

## Data Availability

Due to the qualitative nature of the collected data it has not be linked or uploaded to any publicly archived datasets. If researchers would like access to this data they can approach the corresponding author with a written request. Data regarding the quantitative work on which the accuracy of the training tool should be requested via Lyn.Jones@nbt.nhs.uk. A copy of the semi-structure interview schedule used in this work should be requested via sharding.jb@gmail.com. Qualitative data collected and reporting herein is not available for sharing due to stakeholder views during development that this may impact peoples willingness to participate in the interviews. Study participants did not provide consent for their qualitative data to be uploaded to a repository. If readers would like to apply for access to anonymised data, they can email the corresponding author. This email should; specifying the use to which the data will be put, the time frame of the project and institution which will hold responsibility for the governance of the project that the data is being requested for. Ideally ethical approval will also be provided to cover the requesters project.

## Software availability statement

NVivo 12 ^20^ is a proprietary software. A free open access equivalent to this would be QualCoder available from GitHub ( https://github.com/ccbogel/QualCoder/releases/tag/3.6).

Picture Archiving and Communication System (PACS) is a proprietary software. However, no open access equivalent is available.
