# Supplementary material for: Learning to read FAST MRI: Qualitative interviews with groups experienced reading mammograms
Source: NIHR Open Res. 2025 Apr 24;5:26. Originally published 2025 Mar 20. [Version 2] doi: 10.3310/nihropenres.13817.2 (PMC12130769; doi:10.3310/nihropenres.13817.2)
Supplement: Peer review history

# Reviewer report: Reviewer response for version 2

**DOI:** 10.3310/nihropenres.15175.r35803

**Published:** 2025-06-02

**Authors:** Vivian Youngjean Park

**Affiliations:** 1 Yonsei University College of Medicine, Seoul, South Korea

The widespread implementation of breast MRI screening faces several challenges, including the need to minimize scan time and ensure the availability of adequately trained personnel to interpret a substantially increased volume of studies. Efficient, targeted training will be essential, particularly for readers without prior experience in breast MRI interpretation. This qualitative summary examines the acceptability of a training programme developed for mammogram readers to interpret abbreviated breast MRI, and identifies barriers and facilitators to participation and task performance. The findings offer useful insights for institutions considering similar training initiatives.

 The following comments are intended to enhance contextual clarity, especially for readers less familiar with previous related studies (references 11–13).

  

1. Although the Study Limitations section notes that 34 participants were initially invited, this number (n = 34) should also be reported in the Methods section. The current phrasing in the ‘Training context’ paragraph may be misinterpreted to suggest that only the 14 included participants were invited.
2.  Regarding Capability on Training day, as participants commented on how the training was appropriate for individuals with varying levels of experience, it would be helpful to briefly describe how this aspect was incorporated into the programme. As this may fall outside the main scope of the study, referencing a previous publication with relevant details would aid reader understanding.

Is the work clearly and accurately presented and does it cite the current literature?

Yes

If applicable, is the statistical analysis and its interpretation appropriate?

Not applicable

Are all the source data underlying the results available to ensure full reproducibility?

Partly

Is the study design appropriate and is the work technically sound?

Yes

Are the conclusions drawn adequately supported by the results?

Yes

Are sufficient details of methods and analysis provided to allow replication by others?

Partly

Reviewer Expertise:

breast imaging

I confirm that I have read this submission and believe that I have an appropriate level of expertise to confirm that it is of an acceptable scientific standard.

# Reviewer report: Reviewer response for version 2

**DOI:** 10.3310/nihropenres.15175.r35352

**Published:** 2025-05-05

**Authors:** Jean M Seely

**Affiliations:** 1 University of Ottawa, Ottawa, Ontario, Canada

The authors have responded well to my reviews. I am satisfied with the results.

Is the work clearly and accurately presented and does it cite the current literature?

Yes

If applicable, is the statistical analysis and its interpretation appropriate?

Not applicable

Are all the source data underlying the results available to ensure full reproducibility?

Partly

Is the study design appropriate and is the work technically sound?

Partly

Are the conclusions drawn adequately supported by the results?

Partly

Are sufficient details of methods and analysis provided to allow replication by others?

Partly

Reviewer Expertise:

Breast Imaging - Radiologist

I confirm that I have read this submission and believe that I have an appropriate level of expertise to confirm that it is of an acceptable scientific standard.

# Reviewer report: Reviewer response for version 1

**DOI:** 10.3310/nihropenres.15011.r35042

**Published:** 2025-04-09

**Authors:** Jean M Seely

**Affiliations:** 1 University of Ottawa, Ottawa, Ontario, Canada

This is an interesting summary of qualitative interviews of readers trained to learn Abbreviated breast MRI in a one-day training session. It explored the acceptability of the implementation of developed reader training, and the barriers and facilitators to training programme participation. The interviews summarized with some comments. There are details that are required to assist in the interpretation of the study. Although it is recognized that it was qualitative, the numbers are lacking on how many answered positively, or negatively for each category. The points below would help in the study communication and interpretation.

1. NHSBSP multi-professional mammogram readers, fully qualified to interpret mammograms – the way it is reported suggested that both radiographers and radiologists were trained, but this should be clearly stated. In the previous study, ref 11, the number of radiographers, breast clinicians and radiologists was listed. This would be expected for this study. 
2. Participants attended a single day of face-to-face standardised training and then interpreted a test set of abMRI scans.  Was this done at a single site on the same day for all participants?
3. The authors should clarify if the interviews were done after the test set was administered, or after the training set.  If the former, they should provide the period of time that the test set was delivered subsequently, with the average and range. I believe this is separate to what the authors referred to in the text:  “Following the training, there was an unexpected gap before the participants were able to access the training images (median 3.5 months (range 1.8 – 8.9). “
4. Were participants paid to do the day? It is possible that payment for the radiographers may have biased the outcome, if they were paid to do the training while the radiologists were not. A bit more context on this would be helpful. 
5. What was the timeline for the interviews after the training set or test set and was recall bias avoided? The authors should provide the timeframe for the interviews and should add the limitation of recall bias in the discussion.
6.  It would be quite possible that the selection bias of only 14/37 respondents led to an incorrect or at least incomplete assessment of the results. That should be discussed as a limitation of the study. Do the authors have any ideas what drove the self-selection of the 14 who responded?
7.  The questions asked during the interviews should be provided and a summary of whether standardized interview questions were asked of every participant.

Is the work clearly and accurately presented and does it cite the current literature?

Yes

If applicable, is the statistical analysis and its interpretation appropriate?

Not applicable

Are all the source data underlying the results available to ensure full reproducibility?

Partly

Is the study design appropriate and is the work technically sound?

Partly

Are the conclusions drawn adequately supported by the results?

Partly

Are sufficient details of methods and analysis provided to allow replication by others?

Partly

Reviewer Expertise:

Breast Imaging - Radiologist

I confirm that I have read this submission and believe that I have an appropriate level of expertise to confirm that it is of an acceptable scientific standard, however I have significant reservations, as outlined above.
